# Uptake of Silicon by Sugarcane from Applied Sources May Not Reflect Plant-Available Soil Silicon and Total Silicon Content of Sources

**DOI:** 10.3389/fpls.2017.00760

**Published:** 2017-05-15

**Authors:** Malcolm G. Keeping

**Affiliations:** ^1^South African Sugarcane Research InstituteMount Edgecombe, South Africa; ^2^School of Animal, Plant and Environmental Sciences, University of the WitwatersrandJohannesburg, South Africa

**Keywords:** acid soils, aluminum, calcium silicate, liming, plant stress, silicon uptake, soil pH, thermophosphate

## Abstract

Soils of the tropics and sub-tropics are typically acid and depleted of soluble sources of silicon (Si) due to weathering and leaching associated with high rainfall and temperatures. Together with intensive cropping, this leads to marginal or deficient plant Si levels in Si-accumulating crops such as rice and sugarcane. Although such deficiencies can be corrected with exogenous application of Si sources, there is controversy over the effectiveness of sources in relation to their total Si content, and their capacity to raise soil and plant Si concentrations. This study tested the hypothesis that the total Si content and provision of plant-available Si from six sources directly affects subsequent plant Si uptake as reflected in leaf Si concentration. Two trials with potted cane plants were established with the following Si sources as treatments: calcium silicate slag, fused magnesium (thermo) phosphate, volcanic rock dust, magnesium silicate, and granular potassium silicate. Silicon sources were applied at rates intended to achieve equivalent elemental soil Si concentrations; controls were untreated or lime-treated. Analyses were conducted to determine soil and leaf elemental concentrations. Among the sources, calcium silicate produced the highest leaf Si concentrations, yet lower plant-available soil Si concentrations than the thermophosphate. The latter, with slightly higher total Si than the slag, produced substantially greater increases in soil Si than all other products, yet did not significantly raise leaf Si above the controls. All other sources did not significantly increase soil or leaf Si concentrations, despite their high Si content. Hence, the total Si content of sources does not necessarily concur with a product's provision of soluble soil Si and subsequent plant uptake. Furthermore, even where soil pH was raised, plant uptake from thermophosphate was well below expectation, possibly due to its limited liming capacity. The ability of the calcium silicate to provide Si while simultaneously and significantly increasing soil pH, and thereby reducing reaction of Si with exchangeable Al^3+^, is proposed as a potential explanation for the greater Si uptake into the shoot from this source.

## Introduction

Although silicon (Si) is abundant in the Earth's crust (28.8%) (Wedepohl, 1995), it is not considered an essential element for terrestrial plants other than the Equisitaceae (Epstein, 1994). However, there is now considerable evidence for its role in plant health and ecology (Cooke and Leishman, 2011, 2012), and specifically in mitigating numerous abiotic and biotic stresses, including water and salinity stress, metal toxicities, nutrient imbalance, fungal and bacterial pathogens, and insect herbivores (reviews by Ma, 2004; Datnoff et al., 2007; Liang et al., 2007; Epstein, 2009; Reynolds et al., 2009; Zhu and Gong, 2014; Adrees et al., 2015). Among crop species that accumulate Si to levels >1.0% shoot Si dry mass (Ma and Takahashi, 2002), rice (*Oryza sativa* L.) and sugarcane (*Saccharum* spp. hybrids) have been well-studied, and are capable of removing up to 470 and 700 kg Si ha^−1^ annum^−1^, respectively, on Si-rich soils (Ross et al., 1974; Savant et al., 1997b, 1999; Meena et al., 2014). Yield responses in rice and sugarcane to soil Si amendments have frequently been recorded on the weathered tropical or sub-tropical soils on which they are largely grown (e.g., Oxisols, Ultisols, and organic Histosols) (Cheong and Halais, 1969; Elawad et al., 1982; Yamauchi and Winslow, 1989; Savant et al., 1997b, 1999; Alvarez and Datnoff, 2001; Meyer and Keeping, 2001; Berthelsen et al., 2001b; Tsujimoto et al., 2014). Due to high rainfall and temperatures, such soils have typically been depleted (desilicated) of soluble sources of Si (McKeague and Cline, 1963c; Savant et al., 1997a; Epstein, 2001; Meena et al., 2014), leading to marginal or deficient levels of plant Si in these crops (Savant et al., 1997a, 1999; Meena et al., 2014). Besides being low in essential nutrients, these highly weathered soils are also acidic and may therefore be high in soluble forms of aluminum (Al) (where soil pH_w_ < 5.5) (Sanchez, 1976; Fageria et al., 1988), which in turn can remove soluble Si through reaction to form insoluble hydroxyaluminosilicates (HASs) (Farmer et al., 1979; Doucet et al., 2001; Schneider et al., 2004).

Plants take up Si as monomeric silicic acid (H_4_SiO_4_), the dominant form of Si in soil solution (Epstein, 1994). The solubility of silicic acid in soil solution is strongly pH-dependent and related to its adsorption/desorption reactions on soil colloids. Solubility and concentration in soil solution is highest at low pH and decreases progressively up to a pH of 9.8, the pK_1_ of silicic acid, where the latter dissociates to form H_3_${\text{SiO}}_{4}^{-}$. At this pH, the silicate anion is maximally adsorbed to soil surfaces, especially Al and Fe hydrous oxides, causing the concentration of Si in soil solution to decrease (Beckwith and Reeve, 1963; Jones and Handreck, 1963; McKeague and Cline, 1963a,b; Haynes, 2014; Liang et al., 2015). This relationship between Si solubility and pH is one of the major factors accounting for the loss of Si in weathered, acidic soils, and is exacerbated through intensive, long-term cropping and resulting export of Si from the landscape (Berthelsen et al., 2001b; Sommer et al., 2006; Vandevenne et al., 2012; Haynes, 2014).

A further problem in weathered, acid soils is, as noted above, the occurrence of high levels of exchangeable Al^3+^. Soil acidity and associated Al^3+^ toxicity have long been recognized as significant, and increasing, constraints in sugarcane production in the South African sugar industry (Sumner, 1970; Meyer et al., 1971; Moberly and Meyer, 1975; Schroeder et al., 1994) and indeed wider agricultural production in South Africa (Barnard and du Preez, 2004). For soils under sugarcane production in South Africa, Miles et al. (2014b) showed convincingly that available Si is strongly limited under conditions of high exchangeable H^+^+Al^3+^. Their results indicated that high levels (>40 mg kg^−1^) of soluble Si occurred only where exchangeable H^+^+Al^3+^ levels were below approximately 0.5 cmol_c_L^−1^ and that Al^3+^ is probably a key factor in constraining plant-available Si in acid soils. Differences in available soil Si and pH across regions are strongly reflected in Si uptake by sugarcane, with leaf Si content consistently higher (10–25 g kg^−1^ dry matter) in less acid soils (pH > 6.5), but seldom exceeding the industry threshold (Miles and Rhodes, 2013) of 7.5 g kg^−1^ dry matter in the more weathered acid soils of the coastal and hinterland sugarcane production regions (pH ≤ 5.5) (Van der Laan and Miles, 2010; Miles et al., 2011).

Hence, in these soils there is an urgent need to replenish plant-available soil Si in order to sustain maximum crop production, reduce abiotic stresses (especially water stress and Al toxicity) (Meyer and Keeping, 2001), and as a means to augment plant resistance of more susceptible cultivars to the lepidopteran stalk borer *Eldana saccharina* Walker (Keeping and Meyer, 2006; Kvedaras and Keeping, 2007; Kvedaras et al., 2007; Keeping et al., 2014). Silicon amendment can also reduce infections of brown rust (*Puccinia melanocephala* H. and P. Sydow), which occurs in several rust-susceptible cultivars in South Africa (Ramouthar et al., 2016). With this in mind, recent research efforts on provision of Si for sugarcane production in South Africa have focussed on identifying sources with high plant-available Si, and which can simultaneously correct soil pH and reduce Al toxicity (Rhodes et al., 2013; Keeping et al., 2014, 2017). Calcium (magnesium) silicate, supplied in the form of metallurgical slags, has proven most effective in supplying plant-available Si for sugarcane (Gascho, 2001; Berthelsen et al., 2001a; Meyer and Keeping, 2001; Bokhtiar et al., 2012; McCray and Ji, 2013; Crusciol et al., 2014; Tubana et al., 2016). Keeping et al. (2017) found that alkaline Si sources, such as calcium (Ca) silicate slag, cement, and granulated ground blast furnace slag, produced significantly more plant-available Si and greater plant uptake than sources with little or no pH-corrective capacity, such as potassium (K) silicate, bagasse fly ash, and diatomaceous earth. In line with their ability to increase soil pH, slags and cement also significantly reduced Al saturation, and to an extent equivalent to that of dolomitic lime applied at the same rate (Keeping et al., 2017).

However, other Si sources, such as thermophosphates, sometimes referred to as fused magnesium (Mg) phosphate (see Ma and Takahashi, 2002, p. 18), have also shown significant potential in supplying Si (Korndörfer and Gascho, 1999; Gascho, 2001; Kingston, 2011), as has volcanic rock dust (crushed basalt) when applied to sugarcane on highly weathered soils in Mauritius (D'Hotman De Villiers, 1961, 1962). The latter amendment, applied at rates from 110 to 440 tons ha^−1^, produced cumulative yield responses of between 49 and 90 tons cane ha^−1^ over five crops. Subsequent studies confirmed that the soluble silicon in the basalt accounted for the favorable yield increases (Halais and Parish, 1964). However, previous work has shown that the total Si content of a source, its provision of plant-available Si (as determined by soil tests), and uptake of Si, especially in rice and sugarcane, did not necessarily concur (Gascho, 2001; Ma and Takahashi, 2002; Kingston, 2011; Haynes et al., 2013; McCray and Ji, 2013; Elephant et al., 2016; Keeping et al., 2017). This observation, together with the novel opportunity to investigate several new Si sources (volcanic rock dust, magnesium silicate and slow-release potassium silicate) for sugarcane in South Africa, prompted further study of the relationship between total source (or product) Si content, available (calcium chloride (CaCl_2_) extractable) soil Si following application, and subsequent plant Si accumulation, along with the potential of these sources to reduce acid saturation.

To this end, two trials were conducted using potted sugarcane grown in a low-Si soil, supplied with Si at a single (Trial 1) or two (Trial 2) elemental rates via applications of a Ca silicate slag (Calmasil®, http://www.pbd-lime.co.za/calmasil.htm), thermophosphate (Calsimag-P®), Mg silicate (Prosil Plus®), granular K silicate, volcanic rock (basalt) dust (Turbo-Grow®, www.turbo-grow.co.za), and, in Trial 2, a dolomitic lime control (Table 1). Calmasil is a slag by-product of the stainless industry, while Calsimag-P is manufactured by blending and fusing apatite and serpentine in a furnace, and the resulting amorphous Ca/Mg/P/Si complex is milled to a fine powder and granulated. The Mg silicate (Prosil Plus) is also a serpentine mineral source derived from crushed volcanic rock mined from Colombian batholiths. The K silicate consisted of two types: “Type M” slow release granules that contained compounds of Mg, which imparted a “free running” characteristic to the granules and their slow dissolution in water; “Type MC hardened” slow release granules that contained both Mg and Ca for the same reasons, and had been oven dried at 130–140°C to further reduce their solubility. The study tested the hypothesis that the Si content of these sources (as specified by the supplier) and available soil Si following application, directly affects subsequent plant Si uptake as reflected in leaf Si concentration. More specifically, the thermophosphate and especially volcanic rock dust, Mg silicate and K silicate, with higher total Si contents than the slag (>2-fold higher for the rock dust and 3-fold higher for the K silicate; Table 1), were predicted to produce significantly higher leaf Si concentrations than the latter. Furthermore, the sources were compared with respect to their ability to ameliorate soil acidity and Al toxicity, and to supply Ca and Mg to the soil and plant.

**Table 1:** **Product name (in alphabetical order), supplier (all South Africa based except for Prosil Plus) and silicon content of products used in Trials 1 and 2**.

**Product**	**Supplier**	**Percent Si**
Calmasil (calcium silicate)^a^	PDB Lime (Pty) Ltd., Middleburg, Mpumalanga	10.3
Calsimag-P®^b^	Farmsecure Agri Science, Amanzimtoti, KZN	12.6
Kulu dolomitic lime^c^	Geyser's Fertilizer and Lime, Durban, KZN	0.0
Potassium silicate type M	Tangmere Resources (Pty) Ltd., Uvongo, KZN	30.8
Potassium silicate type MC	Tangmere Resources (Pty) Ltd., Uvongo, KZN	30.8
Prosil Plus WP (magnesium silicate)^d^	AgroMatChem Ltd., Ta'Xbiex, Malta	16.3
Turbo-Grow® (volcanic rock dust)^e^	Turbo-Grow (Pty) Ltd., Wendywood, Gauteng	24.9

*Note that the Kulu dolomitic lime was used as a control (zero Si) and is listed here for the sake of completeness regarding products used*.

a *Calmasil: electric arc furnace slag, 24.8% Ca, 6.0% Mg*.

b *Calsimag-P: granulated thermophosphate, 21.5% Ca, 8.0% Mg, 8.7% P*.

c *Dolomitic lime: 21.0% Ca, 8.1% Mg*.

d *Prosil: 18.1% Mg*.

e *Turbo-Grow: volcanic rock dust: 5.4% Ca, 3.2% Mg (all Turbo-Grow values based on analysis by SGS Lakefield Research, Booysens, South Africa)*.

## Materials and methods

The trials were established in a randomized design in a shadehouse with clear polycarbonate roofing and walls of 40% green shade cloth, over the period November 2013 to May 2014 (Trial 1; 27 weeks) and October 2014 to March 2015 (Trial 2; 22 weeks). Treatments were replicated 12 times in each trial, with one pot in each row of pots comprising a single replicate of each treatment. All pots (total of 84 in Trial 1 and 96 in Trial 2) were filled with soil collected from the same site within a sugarcane field (Field 380, Inanda Farm, 29°37′37″S, 30°56′58″E, KwaZulu-Natal (KZN), South Africa). The soil collected for Trial 1 was taken from an area immediately adjacent to that collected for Trial 2. The soil type was an Inceptisol (Soil Survey Staff, 2006), which in the USA is among the soil orders commonly found in humid and sub-humid regions, and known to have limiting plant-available Si (Tubana et al., 2016). In KZN, the soil consists of gray loamy sands, moderately to strongly acid, with a low level of fertility (Beater, 1970), and is typical of the weathered, acidic, low-Si soils of the rainfed regions of the South African sugar industry, as described earlier. The soil for each trial was collected from the top 15 cm layer within an area of ~400 m^2^, air dried, thoroughly mixed, and passed through a 1 mm sieve. Single samples for analysis were taken from the mixed and sieved bulk soil for each trial before it was placed into pots. The soil properties of each bulk collection are summarized in Table 2. Although the collection site was specifically chosen due to the acid nature of the soil, it was discovered after analysis that the soil for Trial 1 was of a higher pH, higher Ca and clay content, and much lower acid saturation than that for Trial 2 (Table 2). The most likely explanation is that the area from which the soil for Trial 1 had been collected had inadvertently been limed or used as a site for dumping lime by the grower some time—possibly several years —previously. Consequently, control treatments incorporating dolomitic lime were not included in Trial 1 (see below). Fortuitously, these differences between the soil in each trial provided an opportunity to compare Si uptake and effect on soil properties of two sources (Calmasil and Calsimag-P) common to both trials.

**Table 2:** **Characteristics of soil from Inanda Farm (KwaZulu-Natal, South Africa) collected September 2013 from immediately adjacent areas in the same field for Trials 1 and 2**.

**Trial**	**P^a^ (mg L^−1^)**	**K (mg L^−1^)**	**Ca (mg L^−1^)**	**Mg (mg L^−1^)**	**Si (mg L^−1^)**	**Total cations (cmol L^−1^)**	**pH (CaCl_2_)**	**H^+^+Al^3+^ (cmol L^−1^)**	**Acid saturation^b^ (%)**	**Organic matter (%)**	**Clay (%)**
1	60	152	635	47	14	4.5	4.5	0.5	10.6	3.8	13.0
2	67	142	282	32	10	3.4	4.0	1.3	38.8	4.1	16.4

a *Truog analysis*.

b *Acid saturation = [(H + Al)/(H + Al) + Ca + Mg + K + Na] × 100*.

### Treatments and fertilizer

Plastic pots (6.41 L) were filled with 6,700 g of soil and application rates for all Si treatments (products), lime and fertilizers were converted from kg ha^−1^ to g kg^−1^, based on the average (disturbed) soil density (1,215 g cm^−3^) and a top-soil depth of 15 cm; i.e., a soil mass of 1,822,500 kg ha^−1^. For Trial 1, the five Si sources (Calmasil, Calsimag-P, Prosil Plus, K silicate Type M and Type MC) were all applied at product rates (Table 3) intended to provide an elemental Si rate of 300 kg Si ha^−1^ across all treatments. The control was left untreated, as the soil collected for Trial 1 was of a slightly higher pH and lower acid saturation than that for Trial 2 (Table 2). Consequently, control treatments incorporating dolomitic lime were considered unnecessary for Trial 1.

**Table 3:** **Treatments and product rates for Trial 1**.

**Treatment**	**Product rate**	
	**kg ha^−1^**	**mg kg^−1^**
Control	0	0
Calmasil	2,913	1,598
Calsimag-P	2,459	1,349
Prosil Plus	1,840	1,010
K silicate type M	974	534
K silicate type MC	974	534

*Products were applied at rates intended to provide an elemental silicon rate of 300 kg Si ha^−1^ across all treatments except the control, where no product was applied (zero silicon)*.

For Trial 2, Calmasil, Calsimag-P, and Turbo-Grow, plus a dolomitic lime control with zero Si, were each applied at two rates to produce a total of eight treatments (Table 4). The Si sources were applied to provide a lower Si rate (Si 1) of 300 kg Si ha^−1^ and a higher rate (Si 2) of 750 kg Si ha^−1^ (Table 4). The rates for the dolomitic (Kulu) lime control were made equivalent to that of Calmasil, given the similar neutralizing capacity of the two materials (Calmasil = 102.8% of pure CaCO_3_).

**Table 4:** **Treatments, product rates and silicon rates for Trial 2**.

**Treatment**	**Product rate**		**Silicon rate**	
	**kg ha^−1^**	**mg kg^−1^**	**kg ha^−1^**	**mg kg^−1^**
Dolomitic lime 1	2,913	1,598	0	0
Dolomitic lime 2	7,282	3,996	0	0
Calmasil Si 1	2,913	1,598	300	165
Calmasil Si 2	7,282	3,995	750	412
Calsimag-P Si 1	2,459	1,349	300	165
Calsimag-P Si 2	6,148	3,373	750	412
Turbo-Grow Si 1	1,205	661	300	165
Turbo-Grow Si 2	3,012	1,653	750	412

*Products were applied at two rates to provide a lower (Si 1) and higher (Si 2) elemental silicon rate. The lower rate is the same as that used throughout Trial 1 (see Table 3). Dolomitic lime was applied (as a control with zero silicon) at the same product rates as Calmasil due to the equivalent liming capacity of the two products*.

The particle size distributions of the Si sources used in Trials 1 and 2 (Table 5) were determined by shaking 100 mg samples through 8 sieve sizes for 5 min using a Fritsch (Germany) Pulverisette 03502 mechanical vibrator.

**Table 5:** **Particle size distribution of silicon sources used in Trials 1 and 2**.

**Source**	**Particle size distribution (%)**					
	**>5.0 mm**	**5.0–2.0 mm**	**2.0–1.0 mm**	**1.0–0.5 mm**	**0.5–0.2 mm**	**<0.2 mm**
Calmasil	0.0	0.0	0.3	5.8	26.4	67.5
Calsimag-P	0.5	94.3	4.3	0.0	0.0	0.9
Turbo-Grow	0.0	0.0	0.5	3.7	4.2	91.6
Prosil Plus	0.1	0.1	0.5	3.3	5.6	90.4
K silicate type M	0.6	97.0	0.8	0.6	0.4	0.6
K silicate type MC	9.0	75.3	9.5	3.6	1.6	1.0

*Particle size was determined using a Frisch Analysette equipped with King Test laboratory sieves*.

Treatments and fertilizer were simultaneously hand-incorporated into the entire volume of soil following initial moistening with 1,000 ml water pot^−1^. Basal fertilizers were applied as follows: 88 mg nitrogen kg^−1^ as limestone ammonium nitrate (LAN), 66 mg phosphorous (P) kg^−1^ as Ca di-orthophosphate, 2 mg copper kg^−1^ as copper sulfate, and 8 mg zinc kg^−1^ as zinc sulfate. In both trials, 173 mg K kg^−1^ as K chloride was supplied to all treatments except the K silicate treatments in Trial 1, where top-up K at 35 mg kg^−1^ was provided as K chloride. Top dressings of LAN (54 mg N kg^−1^) and K chloride (71 mg K kg^−1^, Trial 1) or K sulfate (Trial 2, 69 mg K kg^−1^ and 28 mg S kg^−1^) were supplied twice monthly after planting.

### Plants

Sugarcane transplants of variety N12 (Anon, 2006) were produced from single-budded setts cut from mature stalks of field-grown cane of the same age and from the same field. Single 1-month old transplants were planted into each pot immediately after the first soil sample (see below). Pots were drip irrigated daily to weekly, depending on moisture demand.

### Soil and leaf analysis

Soil samples were taken with an augur inserted to the base of all pots in each trial at 7 days after application of treatments and again at 7 days after the trials were harvested at age 27 weeks (Trial 1) or 21 weeks (Trial 2). The purpose of the 7-day interval before the first sample was to allow time for equilibration and any sorption of H_4_SiO_4_ to sesquioxides and soil surfaces that may affect measurement of its availability (Babu et al., 2016a). Samples from 3 (Trial 1) or 4 (Trial 2) adjacent replicates of the same treatment were composited to reduce analysis costs. Plant-available soil Si was determined using 0.01 *M* calcium chloride (CaCl_2_) extraction, a widely-accepted method that provides a close approximation of the soil environment (Berthelsen and Korndörfer, 2005; Sauer et al., 2006; Haynes et al., 2013; Miles et al., 2014b; Babu et al., 2016b). Soil analyses were performed by the South African Sugarcane Research Institute (SASRI) Fertilizer Advisory Service (FAS), with Si and P concentrations determined using the ammonium molybdate blue colourimetry (Liang et al., 2015) and Truog methods (Miles et al., 2014a), respectively. All soil analyses in the FAS are performed on a volumetric basis. Only results pertinent to the hypotheses tested in this study and for elements provided by the Si sources are reported, i.e., soil concentrations of Si, Ca, Mg, P, as well as pH and acid saturation.

Leaf sampling was conducted once, at harvest. The third fully unfurled or “top visible dewlap” leaf was removed from the major tillers in each pot; leaf blades were stripped from the midrib and the blades dried, ground and analyzed for their elemental nutrient content by the SASRI FAS. Samples from 3 adjacent replicates of the same treatment were composited to produce sufficient material for analysis. Leaf Si content was determined using the dry ashing and molybdenum blue colorimetry method (Liang et al., 2015). As for soil, only results pertinent to the hypotheses tested and for elements provided by the Si sources are reported, i.e., leaf concentrations of Si, Ca, Mg, P.

### Data analysis

All data were tested for univariate normality (Shapiro-Wilk test) and homogeneity of variance (Bartlett's test), and appropriate transformations (log or square root) applied when these conditions were not met, prior to analysis of variance. Where ANOVA yielded significant differences between treatments, planned comparisons of means were performed using the Holm-Sidak multiple-comparisons test. All analyses were carried out using Genstat 14th Edition.

## Results

### Effects of Si sources on soil properties in pre-plant and post-harvest soil samples—Trial 1

**Figure 1:**
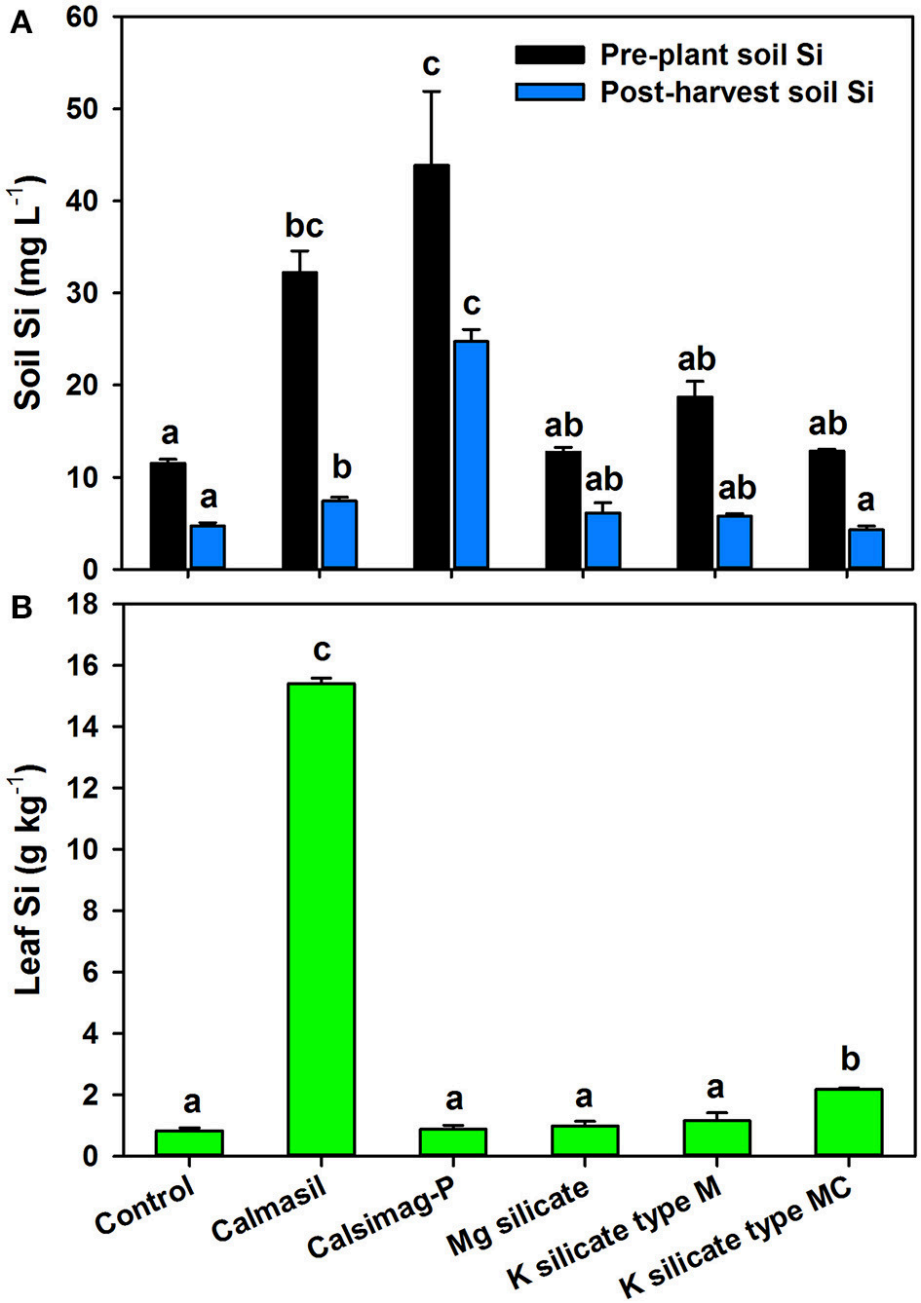
**Silicon concentrations in soil (A)** and third leaf **(B)** following application at rates specified in Table 3 of different silicon sources (represented on X-axis) in Trial 1. The control was untreated. In **(A)**, black bars represent pre-plant soil and blue bars represent post-harvest soil; mean values for bars of the same color and with the same letter/s above them are not significantly different. In **(B)**, mean values for bars with the same letter above them are not significantly different (Holm-Sidak test, *P* < 0.05; ANOVA, *P* < 0.001 for soil and leaf Si). Error bars are standard errors. Mg silicate = Prosil Plus

The soil treatments significantly affected (*P* < 0.001; ANOVA) soil Si concentrations in the pre-plant and post-harvest soil samples. Of the five Si sources applied, only Calmasil and Calsimag-P significantly increased CaCl_2_-extractable soil Si above that of the untreated control in pre-plant and post-harvest samples (Figure 1A). Calsimag-P released greater quantities of Si than Calmasil, but the difference was significant only in the post-harvest soil sample (Figure 1A); this was despite application of Calsimag-P at a lower product rate (Table 3) to compensate for its higher total Si content. Concentrations of extractable Si diminished between the pre-plant and post-harvest soil samples by 59% (control), 77% (Calmasil), 44% (Calsimag-P), 52% (Prosil Plus), 69% (K silicate type M), and 67% (K silicate type MC) (Figure 1A).

The soil treatments significantly affected soil Ca concentrations in the post-harvest sample and Mg and P concentrations in the pre-plant and post-harvest soil samples (Table 6). In the post-harvest treatment, Calmasil supplied significantly more Ca than all other treatments except Calsimag-P. None of the other treatments differed in this respect (Table 6). Calmasil and Calsimag-P both supplied significantly more Mg than the other treatments in pre-plant and post-harvest samples, and Prosil Plus significantly more Mg than the K silicate treatments, which did not differ from the control (Table 6). Calsimag-P increased P significantly compared with the Prosil Plus and K silicate treatments in the pre-plant sample and significantly above all other treatments in the post-harvest sample (Table 6).

**Table 6:** **Soil concentrations of elements (calcium, magnesium, phosphorus) provided by the silicon sources, soil pH and acid saturation in pre-plant and post-harvest samples from Trial 1**.

**Treatment**	**Ca (mg L^−1^)**	**Mg (mg L^−1^)**	**P (mg L^−1^)**	**pH (CaCl_2_)**	**Acid Sat^a^ (%)**
**PRE-PLANT SAMPLE**					
Control	659	58 a	126 ab	4.5 a	8.6
Calmasil	763	83 b	136 ab	4.9 b	2.7
Calsimag-P	681	82 b	203 b	4.8 ab	5.2
Prosil Plus	609	58 a	125 a	4.6 a	8.6
K silicate type M	700	52 a	114 a	4.6 ab	7.0
K silicate type MC	671	55 a	95 a	4.6 ab	8.4
*P*-value	0.84	<0.001	0.002	0.014	0.29
**POST-HARVEST SAMPLE**					
Control	823 a	84 ab	59 a	4.6 a	5.1
Calmasil	1268 b	146 c	54 a	5.3 c	0.2
Calsimag-P	980 ab	131 c	185 b	5.0 bc	1.4
Prosil Plus	804 a	106 b	58 a	4.8 ab	3.7
K silicate type M	820 a	76 a	60 a	4.7 ab	3.7
K silicate type MC	815 a	81 a	57 a	4.6 ab	5.5
*P-*value	0.013	<0.001	<0.001	<0.001	0.15

*Application rates are specified in Table 3; the control was untreated. See Figure 1 for soil silicon concentrations. Values are means (n = 3 for pre-plant, n = 4 for post-harvest). P-values are from ANOVA. Where ANOVA indicates significant differences, means within a column followed by the same letter are not significantly different (Holm-Sidak test, p < 0.05)*.

a *Acid Sat = Acid saturation (see Table 2 for definition)*.

Although the treatments significantly affected soil pH in pre-plant and post-harvest samples, they had no effect on acid saturation (Table 6). In the pre-plant sample, Calmasil raised pH significantly above the control and Prosil Plus, but not the other treatments (Table 6), while in the post-harvest sample, Calmasil increased pH significantly above that of all other treatments except Calsimag-P; the latter was also significantly higher than the control. Prosil Plus and K silicate did not differ from the control (Table 6).

### Effects of Si sources on leaf Si, Ca, Mg, and P concentrations—Trial 1

The soil treatments significantly affected leaf Si concentrations (*P* < 0.001; ANOVA); however, only Calmasil (by 19-fold) and to a much lesser extent K silicate type MC (by ~3-fold) increased leaf Si above the control (Figure 1B). It is clear from Figure 1 that for Calmasil and Calsimag-P leaf Si did not increase in direct relation to the concentration of extractable soil Si.

The treatments had no significant effects on leaf Ca, Mg, or P concentrations (Table 7).

**Table 7:** **Leaf concentrations of elements (calcium, magnesium, phosphorus) provided by the silicon sources applied in Trial 1 at the rates specified in Table 3**.

	**Ca**	**Mg**	**P**
**Treatment**	**g kg**^**−1**^		
Control	1.7	0.9	1.4
Calmasil	1.7	1.0	1.3
Calsimag-P	2.1	1.2	1.3
Prosil Plus	1.9	1.1	1.3
K silicate type M	1.8	0.9	1.3
K silicate type MC	1.9	1.1	1.4
*P*-value	0.61	0.28	0.95

*The control was untreated. See Figure 1 for leaf silicon concentrations. Values are means (n = 4). P-values are from ANOVA*.

### Effects of Si sources on soil properties in pre-plant and post-harvest soil samples—Trial 2

**Figure 2:**
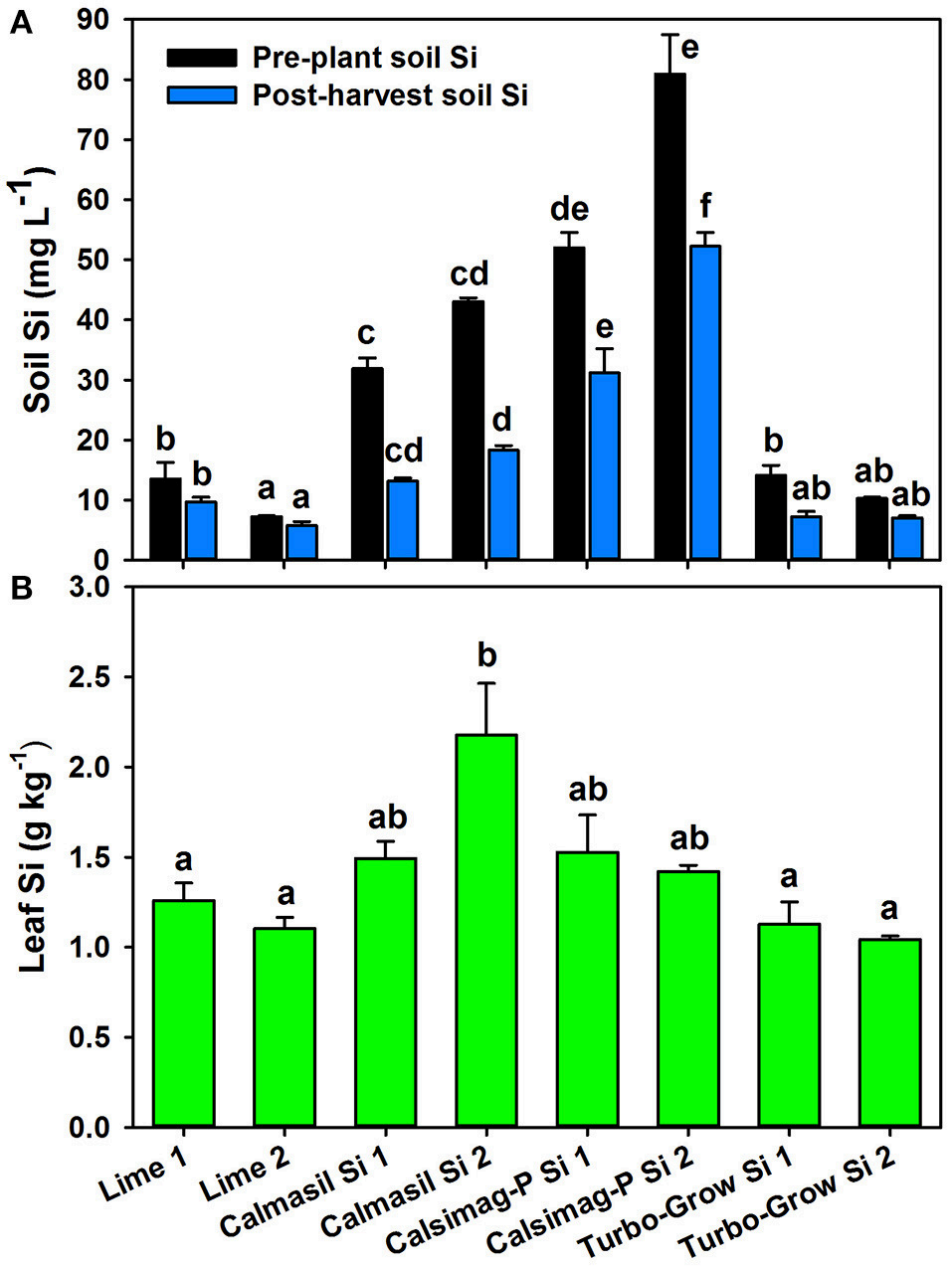
**Silicon concentrations in soil (A)** and third leaf **(B)** following application of different silicon sources (represented on X-axis) at lower and higher rates (Si 1 and Si 2; see Table 4) in Trial 2. Product and silicon application rates are specified in Table 4; the lime control was applied at the same product rates as Calmasil. In **(A)**, black bars represent pre-plant soil and blue bars represent post-harvest soil; mean values for bars of the same color and with the same letter/s above them are not significantly different. In **(B)**, mean values for bars with the same letter above them are not significantly different (Holm-Sidak test, *P* < 0.05; ANOVA, *P* < 0.001 for soil and leaf Si). Error bars are standard errors.

The soil treatments significantly affected (*P* < 0.001; ANOVA) soil Si concentrations in the pre-plant and post-harvest soil samples. Of the three Si sources applied, only Calmasil and Calsimag-P significantly elevated CaCl_2_-extractable soil Si above that of the dolomitic lime control (Figure 2A). Calsimag-P released significantly greater quantities of Si at the higher and lower elemental Si rates than Calmasil at each rate (Figure 2A), even though Calsimag-P was applied at a lower product rate (Table 4) to compensate for its higher total Si content. In the post-harvest sample, Calsimag-P at the lower rate (Si 1) produced significantly higher extractable Si than Calmasil at the higher rate (Si 2) (Figure 2A). Turbo-Grow did not raise extractable soil Si above that of the lime control in both soil samples (Figure 2A). Concentrations of extractable Si decreased between the pre-plant and post-harvest soil samples by 59% (Calmasil Si 1), 57% (Calmasil Si 2), 40% (Calsimag-P Si 1), and 35% (Calsimag-P Si 2), (Figure 2A). Soil Si also decreased over the course of the trial in the Lime 1 (29%), Lime 2 (21%), Turbo-Grow Si 1 (49%), and Turbo-Grow Si 2 (31%) treatments (Figure 2A).

The soil treatments significantly affected soil Ca and Mg concentrations in the pre-plant and post-harvest soil samples, and P in the pre-plant sample (Table 8). In the pre-plant sample, Calmasil Si 2 supplied significantly more Ca than all other treatments and significantly more Mg than all other treatments except Turbo-Grow Si 2 (Table 8). Turbo-Grow Si 2 provided significantly more Ca than dolomitic lime 2 and Calsimag-P Si 2 (Table 8). Compared with lime (which was applied at the same product rates), Calmasil provided 48 and 65% more Ca, and 47 and 63% more Mg, at Si 1 and Si 2, respectively.

**Table 8:** **Soil concentrations of elements (calcium, magnesium, phosphorus) provided by the silicon sources applied at lower and higher rates (Si 1 and Si 2), soil pH and acid saturation in pre-plant and post-harvest samples from Trial 2**.

**Treatment**	**Ca (mg L^−1^)**	**Mg (mg L^−1^)**	**P (mg L^−1^) (mg L^−1^)**	**pH (CaCl_2_)**	**Acid Sat^a^ (%)**
**PRE-PLANT SAMPLE**					
Dolomitic lime 1	638 b	93 ab	153 a	4.5 b	4.8 c
Dolomitic lime 2	829 cd	131 bcd	157 a	5.1 c	1.4 b
Calmasil Si 1	947 de	137 cd	147 a	5.0 c	1.5 b
Calmasil Si 2	1365 f	214 f	154 a	6.0 d	0.5 a
Calsimag-P Si 1	449 a	73 a	234 b	4.4 b	14.4 d
Calsimag-P Si 2	711 bc	166 de	353 c	5.1 c	3.6 c
Turbo-Grow Si 1	660 d	106 abc	168 ab	4.1 a	15.6 d
Turbo-Grow Si 2	1030 e	185 ef	148 a	4.1 a	10.9 d
*P*-value	<0.001	<0.001	<0.001	<0.001	<0.001
**POST-HARVEST SAMPLE**					
Dolomitic lime 1	1025 bcd	179 bc	–	5.1 bc	2.8 b
Dolomitic lime 2	1377 d	237 cd	–	5.9 ef	0.5 a
Calmasil Si 1	1247 cd	176 bc	–	5.4 cd	1.0 ab
Calmasil Si 2	1822 e	259 d	–	6.2 f	0.4 a
Calsimag-P Si 1	668 ab	130 b	–	4.8 ab	8.2 c
Calsimag-P Si 2	985 bc	220 cd	–	5.5 de	1.2 ab
Turbo-Grow Si 1	425 a	49 a	–	4.5 a	21.0 c
Turbo-Grow Si 2	478 a	50 a	–	4.5 a	16.0 c
*P*-value	<0.001	<0.001	–	<0.001	<0.001

*Product application rates are specified in Table 4; the lime control was applied at the same product rates as Calmasil. See Figure 2 for soil silicon concentrations. Values are means (n = 3 for pre-plant, n = 4 for post-harvest). P-values are from ANOVA. Means within a column followed by the same letter are not significantly different (Holm-Sidak test, p < 0.05)*.

a *Acid Sat = Acid saturation (see Table 2 for definition). See text for explanation of missing values for P in post-harvest sample*.

In the post-harvest sample, Calmasil Si 2 still supplied significantly more Ca than all other treatments; however, its supply of Mg was not significantly greater than that of lime 2 or Calsimag-P Si 2 (Table 8). The supply of Ca and Mg by Turbo-Grow at both treatment rates diminished to concentrations significantly lower than that of all other treatments, other than Ca provided by Calsimag-P Si 1 (Table 8). In the pre-plant sample, Calsimag-P Si 2 raised soil P significantly above all other treatments (and 2.3-fold above that of the control) and Calsimag-P Si 1 above all treatments except Turbo-Grow Si 1 (Table 8). Soil P is not presented for post-harvest samples in Table 8, because the FAS laboratory employs different soil P test methods depending on soil pH (Truog for pH ≤ 5.5 and resin for pH > 5.5; Miles et al., 2014a), thus rendering the data from different treatments non-comparable.

The soil treatments significantly affected soil pH and acid saturation in the pre-plant and post-harvest soil samples (Table 8). Calmasil Si 2 raised soil pH significantly above that of all other treatments in the pre-plant and post-harvest samples, with the exception of lime 2 in the post-harvest sample (Table 8). In the pre-plant sample, the *lower* rate of Calmasil (i.e., Si 1) was as effective in correcting pH as the *higher* rate of lime (i.e., lime 2) (Table 8). Although Calsimag-P was applied at a lower product rate than dolomitic lime (Table 4), it nonetheless produced pH levels that were comparable with those of lime at their respective low and high rates (Table 8). Turbo-Grow evidently had little effect in raising pH at the product rates applied, with the pre-plant sample pH elevated by only 0.1 unit and the post-harvest sample by 0.5 unit above that of the untreated bulk soil used in the trial (Tables 2, 5).

Calmasil Si 2 significantly reduced acid saturation percent below that of all other treatments in the pre-plant sample, including lime when compared at the same product rates (Table 8). The reduction of acid saturation (elimination of reactive Al) by Calmasil at the *lower* application rate (Si 1) was not significantly different from that produced by lime at the *higher* application rate (Si 2) (Table 8). Calsimag-P Si 1 and Turbo-Grow Si 1 had similar (and the least) effects on acid saturation, although Calsimag-P Si 2 reduced it to a level not significantly different from that of lime at the lower application rate (dolomitic lime 1) (Table 8). The higher application rate of Turbo-Grow (Si 2) had little effect in reducing acid saturation below that of the lower rate (Si 1) of this product (Table 8). All products however, lowered acid saturation substantially compared with that of the untreated bulk soil (38.8%, Table 2).

Similar differences and similarities between treatments were evident in the post-harvest soil sample; however, it was apparent that acid saturation decreased from pre-plant levels in the lime, Calmasil and Calsimag-P treatments, while that in the Turbo-Grow treatments increased above those of the pre-plant values (values between pre-plant and post-harvest samples were not compared statistically) (Table 8). In the post-harvest sample, acid saturation in the Calsimag-P Si 2 treatment did not differ significantly from that in any of the lime or Calmasil treatments (Table 8).

### Effects of Si sources on leaf Si, Ca, Mg, and P concentrations—Trial 2

The soil treatments significantly affected leaf Si concentration (*P* < 0.001; ANOVA); however, Calmasil Si 2 was the only treatment that significantly increased leaf Si above the dolomitic lime control (Figure 2B). Calmasil Si 2 increased leaf Si 2-fold over that of lime applied at the same product rate (lime 2). Calsimag-P - notwithstanding its substantially greater release of soluble Si into the soil, especially at the higher rate (Figure 2A)-had a small and non-significant effect in raising leaf Si above the lime controls (Figure 2B). Turbo-Grow had no discernible effect on leaf Si content (Figure 2B). As for Trial 1, it is evident for Trial 2 (Figure 2) that leaf Si in the Calmasil and Calsimag-P treatments did not increase in direct relation to the concentration of extractable soil Si.

Leaf concentrations of Ca and Mg were significantly affected by the soil treatments (Table 9). Calmasil Si 1 and Si 2 produced significantly higher leaf Ca than Turbo-Grow Si 2, and Calmasil Si 2 produced significantly higher leaf Ca than lime 2 and Calsimag-P Si 1 and Si 2 (Table 9). For Mg, the only significant difference occurred between Calmasil Si 2 and Turbo-Grow Si 2 (Table 9). The treatments had no significant effect on leaf P concentration (Table 9).

**Table 9:** **Leaf concentrations of elements (calcium, magnesium, phosphorus) provided by the silicon sources applied at lower and higher rates (Si 1 and Si 2) in Trial 2**.

**Treatment**	**Ca**	**Mg**	**P**
	**g kg**^**−1**^		
Dolomitic lime 1	1.5 abc	1.0 ab	1.5
Dolomitic lime 2	1.3 ab	1.1 ab	1.7
Calmasil Si 1	1.6 bc	1.1 ab	1.6
Calmasil Si 2	1.8 c	1.2 b	1.7
Calsimag-P Si 1	1.3 ab	1.0 ab	1.6
Calsimag-P Si 2	1.4 ab	1.1 ab	1.7
Turbo-Grow Si 1	1.6 abc	1.0 ab	1.5
Turbo-Grow Si 2	1.2 a	0.9 a	1.6
*P*-value	<0.001	0.005	0.29

*Product application rates are specified in Table 4; the lime control was applied at the same product rates as Calmasil. See Figure 2 for leaf silicon concentrations. Values are means (n = 4). P-values are from ANOVA. Means within a column followed by the same letter are not significantly different (Holm-Sidak test, p < 0.05)*.

## Discussion

A striking outcome of this study was that although Calmasil had the lowest total Si content (10.3% Si; Table 1), a larger proportion of the Si it provided was taken up by the plant, as it consistently produced the highest leaf Si concentrations in sugarcane. By contrast, sources with high total Si content (K silicate, Prosil Plus, Turbo-Grow; 16.3–30.8% Si; Table 1) produced leaf Si concentrations that were substantially (between 2.2- and 15.4-fold) lower than Calmasil and not significantly different from the lime or untreated controls, other than K silicate type MC (Figures 1B, 2B). The above high-Si-content sources also provided no detectable increases in CaCl_2_-extractable soil Si compared with the controls in both pre-plant and post-harvest samples (Figures 1A, 2A). The low Si provision (even soon after their application) and plant uptake from these sources indicates that they provided little in the way of plant-available Si, despite their high total Si content. Also striking, was the much lower than expected uptake of Si from the Calsimag-P treatments, especially in Trial 1 (Figures 1B, 2B), in direct contrast with this product's substantial and extended provision of extractable soil Si in pre-plant and post-harvest soil samples (Figures 1A, 2A). Although all Si sources were applied at product rates intended to achieve equivalent Si rates of 300 or 750 kg ha^−1^ (Tables 3, 4), the results of the soil analyses clearly show that the total Si content of sources, as stipulated by the manufacturers, cannot be used as a basis for predicting a product's performance in terms of the release of extractable soil Si following its application. These conclusions are borne out by the results of other studies. For example, Haynes et al. (2013) found that a negligible quantity of the very high total Si content (29.1%) of fly ash was in extractable form (using several extractants) compared with steel slag and processing mud, which had the lowest total Si contents but relatively high extractable Si. Korndörfer and Gascho (1999) reported high Si content in steel slag (29%) and Mg silicate (39%), but low availability and uptake (by rice) from these sources compared with wollastonite and thermophosphate. Notably, Elephant et al. (2016) showed from soil incubation studies that Calmasil produced higher concentrations of CaCl_2_-extractable soil Si than Langfos® (crushed sedimentary phosphate rock) and quarry dust (crushed dwyka tillite), with 14.2 and 24.4% total Si, respectively.

A further conundrum with respect to the Si supplying capacity of the sources studied here is the role of particle size. Studies have generally shown that Si availability increases as particle size decreases and surface area of dissolution increases (Medina-Gonzales et al., 1988; Datnoff et al., 1992; Gascho, 2001; Ma and Takahashi, 2002; Haynes et al., 2013). However, in the present study, the materials with the finest particle size (Turbo-Grow and Prosil Plus; Table 5) did not have high Si supplying capacity, while Calsimag-P, a granular product (Table 5), released substantial quantities of Si. The form of Si in the product and its solubility are clearly critical, as emphasized by Kingston (2011), Haynes et al. (2013), Babu et al. (2016b) and Tubana et al. (2016). For example, Babu et al. (2016b) noted that slag is a recently formed polycrystalline material and supplies Si at a relatively fast rate and high concentration, while wollastonite, a geologically formed pure crystalline mineral, releases Si at slower rates and lower concentrations.

The potential of thermophosphates, such as Calsimag-P, to be highly efficacious sources of plant-available Si has been demonstrated in other studies (Gascho and Korndörfer, 1999; Pereira et al., 2004; Kingston, 2011). In line with this, Gascho (2001) pointed out that although the total Si content of thermophosphate may be low compared to certain silicate slags (e.g., electric furnace slag, 18.2% Si), the proportion of soluble Si is high. While Calsimag-P did not significantly raise leaf Si content above controls in the present study, this source has previously produced leaf Si values comparable with those of Calmasil using the same soil and sugarcane variety (Keeping et al., 2017); this indicates that uptake from this source can occur, but may be strongly dependent on specific environmental or soil conditions. Such results support Snyder's (2001) point that laboratory analyses of Si-containing materials can only be used as initial screening procedures to identify promising Si sources, and that glasshouse and field studies of plant uptake are ultimately required to provide certainty about the Si supplying capacity of sources.

In the present study, Calmasil significantly increased soil pH in both trials and above that of the equivalent lime treatments in Trial 2 (Table 8); it also reduced acid saturation in pre-plant and post-harvest soil samples, with significantly greater efficiency than dolomitic lime in Trial 2 (Table 8). Notably, Calmasil Si 2 in Trial 2 was the only Si treatment that raised soil pH(CaCl_2_) well above 5.0 in the pre-plant sample (Table 8), at which point Al would precipitate out (Fageria et al., 1988) and its reaction with Si would be reduced. This emphasizes the value of Ca silicate slags in ameliorating soil acidity and Al toxicity, while also supplying Si, Ca, and Mg (Korndörfer and Gascho, 1999; Meyer and Keeping, 2001; Haynes et al., 2013; Castro et al., 2016; Ning et al., 2016). In Trial 1, pH was not increased above 5.0 in the pre-plant sample or above 5.3 in the post-harvest sample (Table 6). Here, Calmasil was applied at only one rate (2,913 kg ha^−1^, equal to the lower Calmasil Si 1 rate in Trial 2), yet the leaf Si concentration of the Calmasil treatment was substantially higher than that in Trial 2 (10-fold more than Si 1 and 7-fold more than Si 2; Figures 1B, 2B). This was not likely due to differences in available soil Si concentration, which was in fact higher in the Calmasil Si 2 treatment in Trial 2 than in the Calmasil treatment in Trial 1 (Figures 1A, 2A). Possibly the already low acid saturation and H^+^+Al^3+^ levels in the soil used in Trial 1 (Table 2) contributed to greater Si uptake and higher leaf Si levels in this trial, given that the soil had probably been limed by the grower many months or even years prior to its use. In contrast to Calmasil, Calsimag-P did not raise soil pH or reduce acid saturation in Trial 2 (Table 8); this may have lowered its effectiveness in elevating leaf Si content as a result of rapid complexation of released Si with soluble, reactive Al^3+^ (Farmer et al., 1979; Doucet et al., 2001; Schneider et al., 2004; Exley, 2012).

Where Si sources are high in total Si but nonetheless provide little or no plant-available Si to the soil, as in the case of the Mg silicate (Prosil Plus), K silicate (Figure 1A), crushed volcanic rock (Turbo-Grow, Figure 2A), diatomaceous earth or fly ash (Kingston, 2011; Haynes et al., 2013; Keeping et al., 2017) and quarry dust (Elephant et al., 2016), the effects of Al^3+^ in reducing their provision of Si to plants would be largely immaterial. However, the situation for slags may be different. Babu et al. (2016a) pointed out that trace amounts of Al^3+^ reduce the equilibrium solubility of Si due to the co-deposition of these elements as hyrdroxyaluminosilicate (HAS) within the soil environment (see Cocker et al., 1998, for a review of this process). They also argued that the presence of Al^3+^ ions on slag particles can reduce the rate of dissolution of silica and act as catalysts in accelerating the process of polymerization of monomeric H_4_SiO_4_ to colloidal silica, which cannot be taken up by plants. Adsorption of the silicate anion (H_3_${\text{SiO}}_{4}^{-}$) to hydroxides of Al and Fe (sesquioxides) increases at higher pH (especially above pH 9) and is of critical importance in constraining the concentration of Si in soil solution (Beckwith and Reeve, 1963; Jones and Handreck, 1963; McKeague and Cline, 1963b; Kato and Owa, 1996; Tavakkoli et al., 2011; Haynes, 2014). This is especially so when higher rates of calcium silicate slag are applied, wherein the higher pH and concentration of solubilizing Si produced by the slag promotes increasing adsorption of H_3_${\text{SiO}}_{4}^{-}$ to sesquioxide surfaces (Haynes et al., 2013; Babu et al., 2016a). While Calmasil contains on average 1.07% Al, the greatest source of this element in the present study, by a very large margin, would be the acid soil used (Table 2). Notwithstanding the substantial reductions in acid saturation in the Calmasil treatments in both trials (Tables 6, 8), small quantities of native soluble Al^3+^ ions may have reduced equilibrium Si solubility or increased its polymerization in the manner described by Babu et al. (2016a). Under field conditions, where wetting and drying cycles would serve to concentrate Si solubilized from slag, polymerization may be especially important in this regard (Keeping et al., 2013).

The mechanisms discussed above do not, however, satisfactorily account for the low concentrations of Si in leaf tissue from the Calsimag-P treatments, where abundant levels of soluble Si were present in the treated soil (Figures 1A, 2A). This suggests that the Si was either not taken up by the plants or, if taken up, not translocated to the shoot. The substantial reductions in soil Si from pre-plant to post-harvest samples in both trials indicate that appreciable plant uptake occurred and/or that some of the available Si was converted during the course of the trials to forms not readily extractable with 0.01 *M* CaCl_2_. Under conditions of low pH, high acid saturation (and therefore presence of soluble Al^3+^), and high Si concentration in the rhizosphere, it is likely that the plant will simultaneously take up Al^3+^ and H_4_SiO_4_ into the root cells, where they may react and co-precipitate (Hodson and Evans, 1995; Cocker et al., 1998; Hodson and Sangster, 1999). As stated by Hodson (2011), co-deposition *in planta* of Al with Si in solid phytoliths is a relatively widespread phenomenon in higher plants, and in roots Al is often co-deposited with Si in epidermal and cortical cells. An *in planta* mechanism that immobilizes Si and inhibits its translocation from the roots to the shoot, may explain the abundant soil Si levels but low (or relatively low) leaf Si concentrations in the Calsimag-P treatments in this study. On the other hand, the robust liming effect of Calmasil may have been sufficient to reduce solubility and plant uptake of Al^3+^ to the extent that its co-deposition with Si within the plant had a much-reduced effect on translocation of Si to the shoot. As mentioned, the already low acid saturation of the soil used in Trial 1 (Table 6) may have accentuated such an effect and promoted the high leaf Si accumulation in the Calmasil treatment (Figure 1B). Detailed studies of Al and Si co-deposition in roots of sugarcane, such as those performed by Cocker et al. (1997) in wheat and Prabagar et al. (2011) in Norway spruce, may reveal *in planta* interactions between these elements, their possible effects on Si translocation, and the extent to which low shoot Si accumulation is a reflection of reactions within the soil (which affect uptake) or reactions within the plant (which affect translocation).

Attention by sugarcane growers to addressing problems of Si and other nutrient deficiencies is an important step in avoiding plant stress and reducing infestation by sugarcane borer (White and White, 2013; Keeping et al., 2014; Nikpay et al., 2015). The present study emphasizes a further essential step, which is to improve soil health and root growth by reducing soil acidity and Al toxicity through liming and/or calcium silicate provision. With increasing acidification of soils due to long periods of monocropping and intensive use of nitrogenous fertilizers, not only in South Africa but across many tropical and sub-tropical crop-growing regions (Meyer et al., 1998; Barnard and du Preez, 2004; Ma, 2004; Fageria and Baligar, 2008; Van der Laan and Miles, 2010; Marafon and Endres, 2013; Meena et al., 2014), the use of calcium silicate slags presents a valuable substitute for conventional dolomitic limes. As demonstrated in the present study and previous studies, slags also provide Ca and Mg (Table 8); both of these nutrients, along with P, were nutritionally adequate in Trials 1 and 2 (Tables 7, 9) (Miles and Rhodes, 2013). Moreover, slags have the additional advantages of supplying Si, being more reactive than lime (and in the current study more effective on a mass for mass basis), and correcting acidity and eliminating Al^3+^ to a greater soil depth (Korndörfer and Gascho, 1999; Pereira and Cabral, 2005; Bokhtiar et al., 2012; Marafon and Endres, 2013; Haynes, 2014; Castro and Crusciol, 2015; Castro et al., 2016). The results presented here indicate that Si sources (such as thermophosphate) that provide ample soluble Si but have limited liming capacity, may not be effective sources in acid soils due to reaction of their solubilized Si with Al^3+^ within the soil and perhaps to a larger extent within the plant. This underscores the advantages of alkaline Si sources that can simultaneously eliminate Al^3+^ in the rhizosphere and reduce its uptake. Future field studies should focus on means to further eliminate Al^3+^ or prevent its reaction with Si, by combining treatments of slag with gypsum and sources of organic matter, such as crop residues, manure or bagasse (the sugarcane stalk residue remaining after juice extraction). Reduction of sub-soil acidity, and the retention of soil moisture and improved rainwater infiltration associated with higher soil organic matter, are crucial practices in ensuring root health (Thorburn et al., 1999; Bell et al., 2001; Pankhurst et al., 2005; Sumner, 2011, 2012), and may be equally important in augmenting Si uptake from both native and applied sources.

Finally, there has been increasing focus on the importance of recycling of crop residues, which may contain large quantities of amorphous Si in the form of phytoliths (i.e., phytogenic Si), back into soils in an effort to compensate for the large-scale and ongoing removal of Si from agricultural landscapes when crops are harvested (Struyf et al., 2010; Clymans et al., 2011; Guntzer et al., 2012; Keller et al., 2012; Vandevenne et al., 2012; Cornelis and Delvaux, 2016). In sugarcane, the dry leaf matter is an important potential source of Si, as the concentration of Si in dry leaf may reach 3% DM (Van Dillewijn, 1952), following the deposition of amorphous Si to form phytoliths in green leaves (Kaufman et al., 1979; Tripathi et al., 2011). A rainfed crop that yields a total biomass of 80 t ha^−1^ at harvest could produce 16 t ha^−1^ dry leaf matter, as dry leaf matter may account for 20% DM of total biomass (Purchase et al., 2008), potentially providing 480 kg Si ha^−1^, assuming the above 3% Si composition. As in the case for rice (Ma and Takahashi, 1991; Savant et al., 1999; Haynes, 2014; Klotzbücher et al., 2016), this alone could provide much of the Si that would otherwise need to be provided in the form of silicate amendments, and highlights the substantial yearly removal of Si from sugarcane fields effected in the process of crop residue removal and burning. Silicon provision through retention of crop residues should therefore be viewed as a prominent but generally overlooked benefit of crop residue retention along with the many other benefits of this practice (Thorburn et al., 1999; Bell et al., 2001; van Antwerpen et al., 2001; Pankhurst et al., 2005) in sugarcane production.

## Conclusions

Vendors of new Si-bearing materials frequently lay considerable emphasis on the high Si content of their products, without clear evidence as to how much of the total Si is plant-available. Yet this and previous studies have shown that the total Si content of sources is not a reliable indicator of how effective they may be in, firstly, releasing sufficient quantities of plant-available Si into soil solution and, secondly, in facilitating its uptake by the plant. In weathered acid soils dominated by Al and Fe sesquioxides, and which occur across much of the tropical and sub-tropical regions where Si-accumulating crops such as rice and sugarcane are grown, calcium silicate appears to be the most effective source in both respects, as it dissolves readily in an acid soil environment to release silicic acid, and is also an efficient liming agent capable of significantly reducing acid saturation and Al toxicity. The latter factor may be critical in constraining reaction of available Si with Al^3+^, either in the soil or in the plant roots, and thereby maximizing Si uptake and translocation to the shoot. Other sources with high Si content either provide very small quantities of soluble Si or, if they do provide adequate Si, may have limited or no liming capacity; consequently they are unable to counteract the direct toxic effects on Al^3+^ on roots or its reaction with silicic acid. As a result of these properties and their provision of ample Ca and Mg, calcium silicate slag appears still to offer the most effective and affordable Si source for sugarcane growers, at least in the acid, sandy soils of the dryland production regions of the South African sugar industry. However, attention should also be directed toward practices that promote recycling of phytogenic Si back into soils, principally through retention of crop residues and preservation of soil organic matter, which in themselves may also promote uptake of Si from silicate slags.

## Author contributions

MK conceived and conducted the research, performed the statistical analyses, and wrote the manuscript.

## Funding

Funding and infrastructural support for the research was provided by the South African Sugarcane Research Institute (SASRI), and additional financing of research expenses by the Incentive Funding Programme for Rated Researchers of the National Research Foundation (NRF), South Africa (Grant No. IFR2011040700004).

### Conflict of interest statement

The author declares that the research was conducted in the absence of any commercial or financial relationships that could be construed as a potential conflict of interest.
